# Quantitative Analysis of Early Retinal Changes and OCT Parameters in Diabetic Subjects with and Without Retinopathy

**DOI:** 10.3390/diagnostics15040451

**Published:** 2025-02-13

**Authors:** Sulaiman Aldakhil, Naveen Challa, Saja A. Alhoshan, Foziyah Abohaimed, Bashair N. Alnasser, Hana A. Almuhawas, Saif AlObaisi, Saif H. Alrasheed

**Affiliations:** 1 Department of Optometry, College of Applied Medical Sciences, Qassim University, Buraidah 51452, Saudi Arabia; s.dakhil@qu.edu.sa (S.A.); n.challa@qu.edu.sa (N.C.);; 2 Department of Ophthalmology, King Abdulaziz Medical City, National Guard Health Affairs, Riyadh 11426, Saudi Arabia; 3 King Abdullah International Medical Research Center, Ministry of National Guard Health Affairs, Riyadh 11481, Saudi Arabia; 4 Department of Ophthalmology, Armed Forces Hospital, Armed Forces Medical Services, Al-Kharj 16274, Saudi Arabia; 5 King Saud Bin Abdulaziz University for Health Sciences, Riyadh 11481, Saudi Arabia

**Keywords:** superficial capillary plexus vessel density, quality of life, diabetes mellitus, diabetic retinopathy, risk factor, Saudi Arabia

## Abstract

**Aim:** The aim of this paper is to assess the changes in optical coherence tomography angiography (OCTA) parameters among normal individuals and for type 2 diabetes mellitus (DM) patients, with and without retinopathy, in the adult Saudi population. **Methods**: This was a prospective cross-sectional study; subjects were divided into four groups. Group 1, the control group, consisted of 40 eyes from normal healthy individuals, while the other three groups included subjects diagnosed with type 2 DM at various stages of retinopathy. All subjects’ OCT and OCTA images were acquired using a swept-source OCT (DRI Triton, Topcon, Inc., Tokyo, Japan). Parameters collected included superficial capillary plexus (SCP) vessel density (VD), foveal avascular zone (FAZ), macular thickness (MT), ganglion cell layer (GCL) thickness, and retinal nerve fiber layer (RNFL) thickness at central and perifoveal locations. OCTA acquisition included a 4.5 × 4.5 mm scan to measure FAZ and SCP VD, with the FAZ manually mapped onto OCTA images at the SCP. **Results**: There was a significant decrease in SCP VD (*p* < 0.05) in all quadrants except the central as the severity of diabetes increased. SCP VD was considerably lower in DM patients without retinopathy compared to controls. Additionally, the FAZ area exhibited a significant increasing trend as the severity of diabetic retinopathy (DR) increased. Regression analysis showed a significant decrease in RNFL thickness (*p* < 0.01) and GCL thickness (*p* < 0.01) in the nasal quadrant as DR severity increased, even after adjusting for age, gender, and mean arterial pressure. Furthermore, SCP VD showed a significant negative correlation with both the duration of DM and contrast sensitivity. **Conclusions**: OCT and OCTA parameters were significantly different between the control and diabetic patients with and without DR. The observed microvascular and contrast sensitivity alterations may precede detectable DR damage or changes in visual acuity.

## 1. Introduction

Diabetic retinopathy (DR) is a vascular disease that affects retinal blood vessels because of long-term complications of diabetes mellitus (DM). It is considered a major complication in working-age groups [1]. DR causes structural changes to the retinal blood vessels, thereby affecting the blood–retinal barrier [2]. Thus, it leads to increased permeability of these microvessels, influencing the retinal layers function [3,4]. Therefore, DR results in decreased vision and significantly impacts visual function, potentially leading to severe visual impairment and blindness [2]. DR has also been associated with neuroretinal deficits even in its early stages and is linked to numerous neurodegenerative changes, such as the loss of ganglion cell bodies, neural apoptosis, and a reduction in thickness of the inner retinal layers [5,6,7,8,9,10].

Recently, the availability of advance imaging and diagnostic tools such as optical coherence tomography (OCT) and optical coherence tomography angiography (OCTA) has improved the early detection of retinal changes before any visible clinical signs [11,12,13,14,15,16]. OCT allows us to perform a high-resolution quantitative analysis of the retinal tissue [17]. It is also very useful in diagnosing and monitoring various retinal disorders by providing the measurement of overall retinal thickness in general and all individual retinal layers [18,19]. Recent studies have found various vascular changes in different stages of DR, including a progressively increasing average vascular diameter, gradually decreasing capillary density, and gradually branching complexity [20,21,22].

The World Health Organization (WHO) has reported that Saudi Arabia has the second highest number of diabetic patients globally [23]. DR causes blindness in approximately 10,000 people with diabetes per year [2]. In Saudi Arabia, the prevalence of DR has increased dramatically in recent decades [24]. It has been reported that the prevalence of DR in Saudi diabetic patients has been reported to vary from 19.7% to 45.7%, with the percentage of those with vision-threatening DR ranging from 4.5% to 17.5% [25,26]. Therefore, early detection and control of DR are essential. Understanding the early undetectable signs before any visual function deterioration occurs may help in the design of a better treatment and follow-up plan. Thus, this study aimed to assess changes in OCT and OCTA parameters in normal and diabetic patients, with and without DR, in the adult Saudi population.

## 2. Methods and Materials

This prospective cross-sectional study included 123 subjects aged between 30 and 60 years. The participants were divided into four groups. A non-probability sampling method was used to select 123 subjects. Group 1, the control group, comprised 40 eyes from normal healthy individuals. Group 2, consisting of 43 eyes, including DM patients without retinopathy. Group 3, with mild non-proliferative diabetic retinopathy (NPDR), comprised 17 eyes. Group 4, with moderate NPDR, included 23 eyes. All individuals were invited to participate voluntarily in this study. Ethical approval was obtained from King Abdullah International Medical Research Center. The study only included type 2 DM patients, as diabetic patients were defined in accordance with WHO guidelines, which include criteria such as fasting plasma glucose levels ≥ 126 mg/dL, 2 h plasma glucose levels ≥ 200 mg/dL, or the current use of antidiabetic medication. Participants in the control group exhibited normal glycemic values. All subjects with intraocular pressure (IOP) greater than 21 mmHg, myopia or hyperopia greater than 6.00 diopters, best-corrected visual acuity less than 0.5 LogMAR, retinal edema, or media opacities were excluded from this study.

Additionally, subjects diagnosed with proliferative DR were excluded. Scans with poor picture quality were excluded based on the following criteria: (i) the presence of retinal edema, (ii) weak local signal or low clarity, (iii) poor fixation resulting in a double vascular pattern or motion abnormalities, and (iv) macular segmentation errors.

Collected data included demographic information for all subjects, including age, systemic history (recent systolic and diastolic hypertension, recent fasting blood glucose levels, and glycated hemoglobin (HbA1c)), and ocular history. A comprehensive eye examination was conducted which included measurements of best-corrected visual acuity with a Snellen chart and a contrast sensitivity test using Sloan’s letters chart that was taken with correction at 40 cm. Additionally, an anterior segment examination was performed using slit-lamp biomicroscopy. A dilated fundus examination was conducted with a direct ophthalmoscope and a 90D lens, focusing on evaluating the foveal area and peripheral retina.

All participants underwent OCT and OCTA imaging using a swept-source OCT device (DRI Triton, Topcon, Inc., Tokyo, Japan). Images were obtained with an acquisition rate of 100,000 A-scans per second and a center wavelength of 1050 nm, yielding an 8 µm axial resolution in tissue. A radial scan measuring 6.0 × 6.0 mm, centered on the fovea, was used to measure macular thickness (MT), retinal nerve fiber layer (RNFL) thickness, and ganglion cell layer (GCL) thickness at the central, superior, inferior, temporal, and nasal regions of the macula, following the ETDRS (early treatment diabetic retinopathy Study) grid. Measurements of RNFL and GCL thickness were automatically obtained from the same 6.0 × 6.0 mm macular radial scan using the instrument’s software.

OCTA acquisition involved a 4.5 × 4.5 mm scan for measuring the foveal avascular zone (FAZ) and assessing the vessel density (VD) in the superficial capillary plexus (SCP). The FAZ area was measured by manually mapping the area along the innermost capillaries on OCTA images at the SCP. The device automatically quantified the retinal vascular density map at the SCP in the 4.5 × 4.5 mm scan. An inbuilt segmentation algorithm provided by the OCT device automatically detected the SCP, located between 2.6 µm below the internal limiting membrane and 15.6 µm below the junction between the inner plexiform and inner nuclear layers (IPL/INLs).

### Statistical Analysis

All data were initially entered into Excel and subsequently transferred to SPSS (IBM, Armonk, NY, USA, version 27.0) for further analysis. Descriptive variables such as age, mean arterial pressure (MAP), the duration of diabetes (in years), fasting blood glucose (FBG), and HbA1c were reported as mean, standard deviation, and 95% confidence intervals. Pearson correlation coefficient was used to assess correlations between the superficial capillary plexus vessel density (SCP VD) and age, HbA1c, and the duration of diabetes. The effects of DR severity, SCP VD, MT, RNFL thickness, and GCL thickness across various regions of the macula were examined using a linear regression model adjusted for age, gender, and MAP. The three groups of DM patients were compared to the control group to investigate DR severity as a dichotomous variable. Linear regression was employed to examine the effects of DR severity on FAZ, and all figures were generated using the SPSS software. Statistical significance was defined as a *p*-value of less than 0.05.

## 3. Results

The study included four groups. A total of 40 control subjects, 43 diabetic subjects without DR, 17 subjects with mild NPDR, and 23 subjects with moderate NPDR. The mean age of the control, DM without DR, mild NPDR, and moderate NPDR groups were 43.86 ± 8.57 years, 52.14 ± 6.17 years, 51.65 ± 7.33 years, and 53.52 ± 5.66 years, respectively. General descriptive statistics of the four groups are presented in Table 1.

The age of the control group was significantly different from that of all other groups (*p* < 0.01). MAP was significantly different (*p* < 0.01) among subjects with DM without retinopathy compared to the other groups. Contrast sensitivity was significantly different between the control group and the NPDR group (*p* < 0.01); however, there were no significant differences among the other groups. Significant variations in the duration of DM were observed among the different patient groups (*p* < 0.01). There was no significant difference (*p* > 0.05) in mean spherical equivalent among the four groups.

### 3.1. Correlation Between VD and FAZ and HbA1c, MAP, and Contrast Sensitivity

The results of the Spearman correlation analysis, presented in Table 2, demonstrate several significant relationships between various variables. Nasal SCP VD is significantly negatively correlated with both the duration of DM and contrast sensitivity. Specifically, the correlation coefficient for nasal SCP VD and the duration of DM is −0.36 (*p* < 0.01), indicating a negative correlation. Similarly, the correlation between nasal SCP VD and contrast sensitivity yields a coefficient of −0.26 (*p* < 0.01). Temporal SCP VD also shows significant negative correlations with the duration of DM (r = −0.24, *p* < 0.05) and contrast sensitivity (r = −0.27, *p* < 0.01). However, SCP VD did not show significant correlations with Hb1Ac or MAP in any quadrant, suggesting that these variables may not be directly related to SCP VD in this context.

FAZ exhibits a significant positive correlation with contrast sensitivity (r = 0.21, *p* < 0.05). No significant correlations were found between FAZ and Hb1Ac or MAP.

### 3.2. Comparison of OCTA Parameters Among the Groups

**Figure 1:**
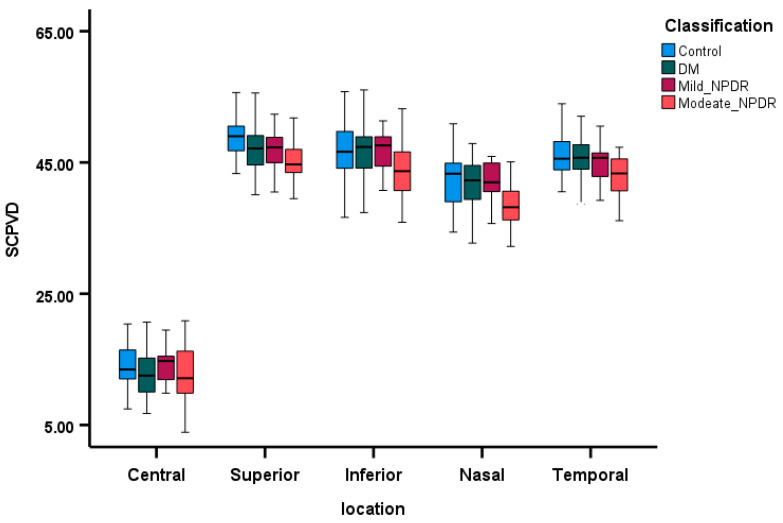
The comparison of SCP VD across the four groups, with box plots depicting the median and 95% confidence intervals, delineated within the five quadrants. SCPVD—superficial capillary plexus vessel density.

The DM patients showed lower SCP VD compared to the control patients, particularly as the severity of DR increased. The box plots in Figure 1 illustrate this difference in SCP VD across the four groups. After adjusting for age, gender, and MAP, it was found that SCP VD was notably reduced in DM patients compared to controls, with a significant decrease in VD in all quadrants except the central quadrant, as detailed in Table 3.

**Figure 2:**
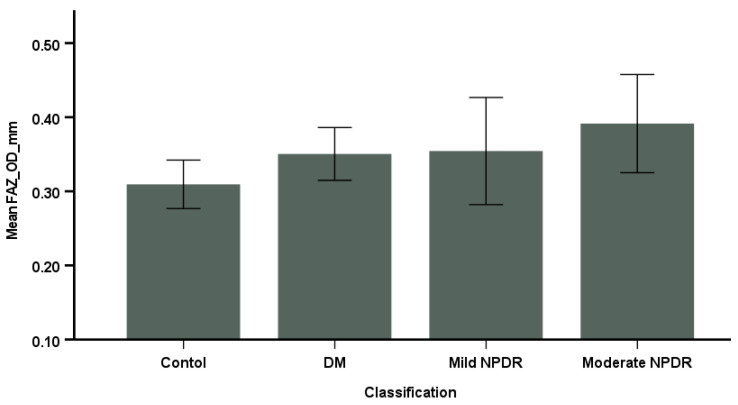
The comparison of the mean foveal avascular zone (FAZ) area across the four groups, presented through bar graphs displaying the mean and 95% confidence intervals.

Additionally, diabetic patients exhibited a significantly larger FAZ area compared to controls, with a trend indicating that the FAZ area increased with the severity of DR, as depicted in Figure 2. Furthermore, there was a significant negative correlation (r = −0.72, *p* < 0.01) between the FAZ area and central SCP VD.

### 3.3. Comparison of OCT Parameters Among the Groups

**Figure 3:**
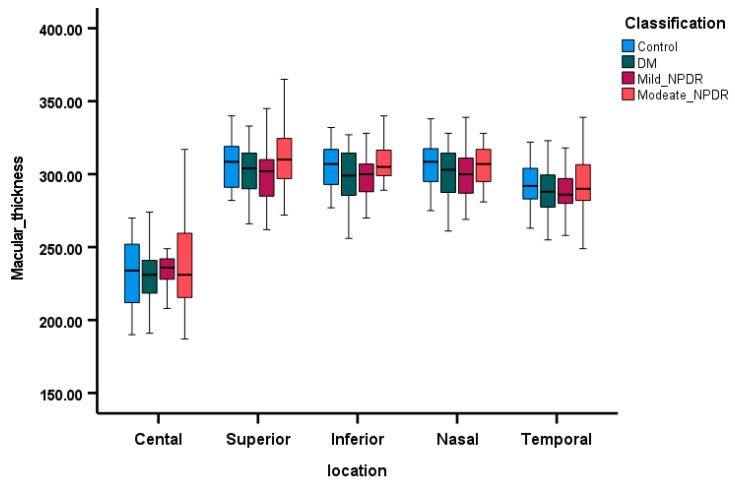
The comparison of macular thickness (µm) across the four groups, utilizing box plots to represent the median and 95% confidence intervals, depicted within five quadrants.

**Figure 4:**
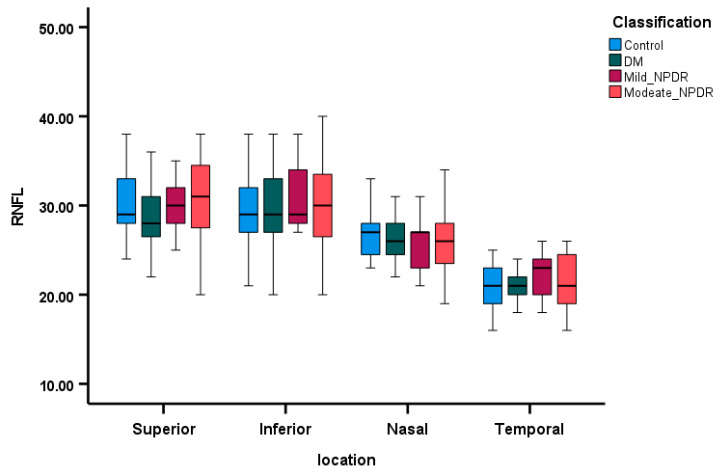
The comparison of RNFL thickness (µm) across the four groups, utilizing box plots to represent the median and 95% confidence intervals, depicted within four quadrants.

**Figure 5:**
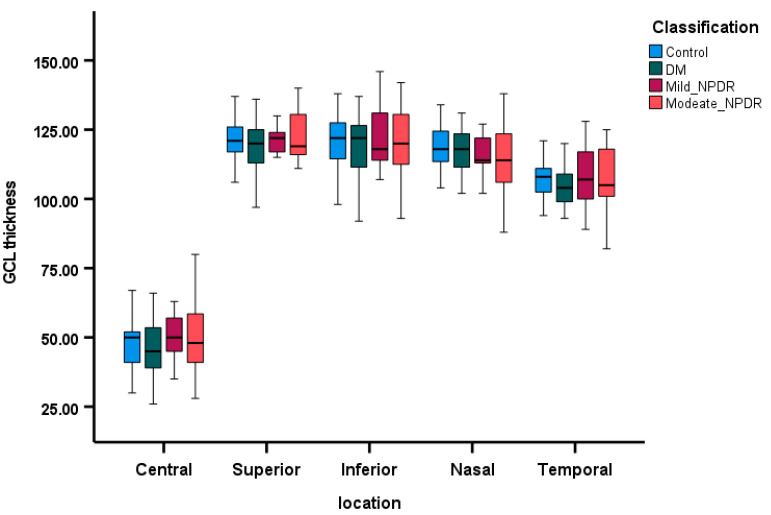
The comparison of GCL thickness (µm) across the four groups, utilizing box plots to represent the median and 95% confidence intervals, depicted within five quadrants.

OCT parameters are displayed as box plots in Figure 3, Figure 4 and Figure 5, illustrating the median MT, median RNFL thickness, and median GCL thickness in the superior, inferior, nasal, and temporal areas of the four groups. Patients with moderate NPDR exhibited thicker MT and RNFLs compared to controls, while patients in the DM group without retinopathy had thinner MT, thinner RNFL, and thinner GCL across all quadrants as depicted in Figure 4 and Figure 5. The regression analysis (Table 4) indicates a significant decrease in RNFL thickness (*p* < 0.01) and GCL thickness (*p* < 0.01) along the nasal quadrant with increasing severity of DR, after adjusting for age, gender, and MAP.

### 3.4. Correlations Between SCP VD and Retinal Layers Thickness

In both the control and diabetic groups, a strong positive correlation was observed between VD in SCP and macular thickness in the central area and between SCP VD and GCL thickness (Table 5). However, the relationship between SCP VD and the other quadrants was found to be weak.

## 4. Discussion

In this prospective cross-sectional study, the early impact of DM on retinal layers was analyzed in comparison with healthy individuals. Diabetic patients exhibited lower retinal SCP VD and a larger FAZ area compared to their healthy counterparts, even in the absence of DR. SCP VD notably decreased as DR severity increased. Moreover, MT, RNFL thickness, and GCL thickness decreased in the very early stages of diabetes without retinopathy compared to healthy individuals. Additionally, contrast sensitivity across all DM groups was significantly lower than in the control group. Linear regression models were employed to explore the effects of DR severity on various retinal parameters, revealing significant relationships between nasal and temporal SCP VD with the duration of diabetes and contrast sensitivity. These findings underscore OCTA as a crucial quantitative technique for the early identification of DR, which could be valuable in establishing effective monitoring and follow-up plans for patients.

The study findings suggest that there are differences in SCP VD in diabetic patients compared to the control group. While previous studies [21,27,28,29,30,31,32] have reported a significant decrease in SCP VD in diabetic patients, other have found no significant difference in SCP and the deep capillary plexus (DCP) VD between diabetic subjects and controls [33,34]. There is also a study that reported a significant decrease in DCP VD but not in SCP VD [35]. In this study, SCP VD in the central (foveal) region was lower in the diabetic participants compared to controls, although this difference did not reach statistical significance, and both groups exhibited normal VA. This implies that a reduction in SCP VD may need to reach a certain threshold to impact VA or could reflect functional vision loss related to DCP VD. It is noted that retinal VD serves as a valuable metric for evaluating visual function in the macular area, with a decrease in VD potentially leading to a decline in VA [36]. Despite normal VA in both groups in our findings, there was a significant difference in contrast sensitivity between the control and diabetic groups, indicating that changes in VD may have a direct or indirect impact on visual function.

Previous research has utilized OCTA to assess the FAZ area in subjects with and without DR. However, findings across studies have shown variability, with some reporting a significantly larger FAZ area in DR subjects compared to controls [30,31,37,38], while others found no significant difference [34,39,40]. Our study results reveal an increased FAZ area among DM subjects compared to controls, with a trend indicating that the FAZ area increases with DR severity. However, a significant difference in the FAZ area was only observed between controls and subjects with NPDR. The considerable interindividual variability in the FAZ area among age-matched groups, both healthy and diabetic, may contribute to the disparities observed in these studies.

It is assumed that higher levels of HbA1c and longer durations of DM would result in more severe microvascular changes in the retina. The result of our study indicates a significant negative correlation between the duration of DM and both nasal and temporal SCP VD. This suggests that as the duration of DM increases, the VD in these areas decreases, consistent with findings from previous research [28,41]. Conversely, SCP VD did not show a significant correlation with HbA1c levels, which aligns with earlier studies [3,21,28].

The findings of the present study reveal a notable negative correlation between nasal RNFL thickness and the severity of DR. Studies from various populations [42,43,44,45,46,47] have demonstrated significant differences in RNFL thickness between healthy subjects and diabetic subjects both with and without DR. One study found significant differences in superior and inferior RNFL thickness between controls and diabetic subjects [42], and others have reported a significant difference in RNFL thickness across all quadrants between control and diabetic subjects [43,44]. However, some studies have reported no significant differences in RNFL thickness in all quadrants between controls and diabetic subjects without DR [47,48].

Among these studies, the nasal RNFL has often appeared less affected [42,43,44,45,46,47], likely due to the sparse distribution of retinal fibers in that region. However, the present study diverges from this trend by revealing a significant reduction in nasal RNFL thickness with increasing severity of DR. This is an unexpected finding, and the underlying cause remains unclear.

Numerous studies have evaluated GCL thickness among healthy individuals and diabetic subjects, both with and without retinopathy. Some studies have reported GCL thinning in DM subjects compared to healthy controls [49], consistent with the findings of the current study where GCL thickness exhibited significant decreases. Others observed a difference in GCL thinning in the perifoveal region among diabetic subjects with early retinopathy compared to controls [49]. These findings suggest that neuroretinal changes may occur in the very early stages of diabetes, potentially preceding visible signs of DR. This has been supported by previous researchers, indicating that neuroretinal damage may play a role in the pathogenesis of DR [50].

The Singapore Eye Study demonstrated that macular thickness did not exhibit a significant difference between non-diabetic and diabetic subjects with no or mild DR. However, diabetic participants with moderate or severe DR showed thicker foveal and temporal outer macula compared to those with no or mild DR [51]. Similarly, another study found significant differences in foveal and parafoveal thickness between diabetic subjects and controls [52]. In contrast, the current study did not find any significant difference in macular thickness across all quadrants between normal and diabetic subjects after adjusting for age, gender, and MAP.

Although the current study highlights the utility of OCTA parameters in the early detection of DR, it has inherent limitations. This study showed significant differences in age between the groups, which may influence the OCTA and OCT parameters. However, a previous study has reported that age has minimal effects on VD below 60 years [53]. Since all the participants in the current study were below 60 years old and the study controlled for age in the analysis, this limitation is somewhat mitigated.

Additionally, while there are reports suggesting the role of DCP VD in detecting the early stages of DR, the current study limited the analysis to SCP VD. Furthermore, the gender ratio was significantly different between the groups. Nevertheless, we utilized a multiple regression model to adjust for gender.

While numerous studies have examined OCT parameters in diabetic patients, our study stands out by focusing on a Saudi Arabian population and including four groups. This allowed us to analyze progressive retinal changes, such as reductions in SCP VD and GCL thickness in early diabetes and the gradual enlargement of the FAZ area with increasing DR severity.

By addressing an underrepresented population, our findings provide valuable insights into potential regional or ethnic variations in retinal changes, contributing to the broader understanding of DR and emphasizing the need for diverse population studies to refine clinical approaches.

## 5. Conclusions

In conclusion, this study found significant correlations between SCP VD and factors such as age, HbA1c levels, and the duration of diabetes. The results also showed a significant thinning in RNFL and GCL thickness in the very early stages of DM, suggesting that neuroretinal changes may occur at the onset of diabetes, even before any sign of DR. Notably, there was a significant difference in contrast sensitivity between the diabetic and control groups, despite both having normal VA. This indicates that alterations in VD may impact visual function either directly or indirectly. Furthermore, microvascular and contrast sensitivity alterations may precede detectable DR damage or changes in VA, suggesting that OCTA and contrast sensitivity could be more sensitive indicators than VA and clinical examination for the early detection of retinal changes. Further research involving larger sample sizes is required to confirm these findings.

## Figures and Tables

**Table 1:** General descriptive data.

Parameter	Control	DM	Mild NPDR	Moderate NPDR	*p* Value
Numbers	40	43	17	23	
Gender					
Male	15 (38%)	11 (25%)	9 (39%)	7 (41%)	
Female	25 (62%)	32 (75%)	14 (61%)	10 (59%)	
Age	43.67 ± 8.57	52.14 ± 6.13	51.65 ± 7.39	53.52 ± 5.66	<0.01
Contrast sensitivity (OU)	2.58 ± 1.15	3.36 ± 1.58	3.32 ± 1.27	4.13 ± 1.55	<0.01
MAP in mm Hg	87.88 ± 9.72	94.98 ± 9.62	86.74 ± 12.11	85.98 ± 10.74	<0.05
Duration of Diabetes (years)	-	8.55 ± 5.53	15.64 ± 7.92	16.48 ± 8.92	<0.01
FBG	5.17 ± 0.69	8.00 ± 3.20	9.80 ± 3.99	11.68 ± 5.18	<0.01
HBA1c	5.39 ± 0.42	7.04 ± 1.32	8.00 ± 1.71	8.75 ± 1.52	<0.01

DM: diabetes militias, NPDR: non-proliferative diabetic retinopathy, MAP: mean arterial pressure, FBG: fasting blood glucose, and HBA1c: glycated hemoglobin.

**Table 2:** Spearman correlation coefficient between SCPVD at various quadrants and diabetes mellitus duration, MAP, Hb1Ac, and contrast sensitivity.

	SCPVD_C	SCPVD_S	SCPVD_I	SCPVD_N	SCPVD_T	FAZ
Duration of DM	0.01	−0.17	0.15	−0.36 **	−0.25 *	−0.02
MAP	−0.10	0.01	0.04	0.10	−0.07	0.07
Hb1Ac	−0.01	−0.14	−0.03	−0.16	−0.14	0.09
Contrast sensitivity	−0.24 **	−0.13	−0.08	−0.26 **	−0.27 **	0.21 *

* Suggest that correlation is significant at the 0.05 level, and ** suggest that correlation is significant at the 0.01 level. C: central, S: superior, I: inferior, N: nasal T: temporal, FAZ: foveal avascular zone SCP: superficial capillary plexus, VD: Vessel density. DM: diabetes mellitus, and MAP: mean arterial pressure.

**Table 3:** Linear regression model showing the correlation between vessel density and the severity of diabetic retinopathy.

OCT Parameter	Regression Co-Efficient			
	Severity of DR	Reference = Control		
		No DR	Mild NPDR	Moderate NPDR
FAZ	0.03 *	0.03	0.05	0.09 *
SCPVD_OD_VD_C	−0.21	−1.37	−0.37	−1.38
SCPVD_OD_VD_S	−0.91 *	−1.25	−1.08	−3.06 *
SCPVD_OD_VD_I	−0.95 *	−0.74	0.08	−3.40 *
SCPVD_OD_VD_N	−0.92 *	−0.86	0.02	−3.31 *
SCPVD_OD_VD_T	−0.96 *	−0.37	−0.86	−3.02 *

Values were adjusted for age, gender, and mean arterial pressure. *p* < 0.05 is significant and shown with the * sign. NPDR: none-proliferative diabetic retinopathy, DR: diabetic retinopathy, OD: right eye, C: central, S: superior, I: inferior, N: nasal T: temporal, FAZ: foveal avascular zone, SCP: superficial capillary plexus, and VD: vessel density. The control group is used as a reference cohort.

**Table 4:** Linear regression model showing the correlation between OCT parameters and the severity of diabetic retinopathy.

OCT Parameter	Regression Co-Efficient			
	Severity of DR	Reference = Control		
		No DR	Mild NPDR	Moderate NPDR
OD_MT_C	1.46	−7.24	−6.70	4.46
OD_MT_S	−0.95	−1.70	−4.34	−2.35
OD_MT_I	−2.29	0.58	−5.15	−5.75
OD_MT_N	−2.35	1.17	−7.80	−4.89
OD_MT_T	2.02	0.30	−3.54	7.89
OD_RNFL_S	0.17	−1.62	0.40	0.14
OD_RNFL_I	0.40	0.30	1.29	1.00
OD_RNFL_N	−0.51 *	−0.12	−1.09	−1.37
OD_RNFL_T	0.02	−0.50	0.44	−0.24
OD_GCL_C	0.21	−2.46	0.85	−0.41
OD_GCL_S	−0.07	−2.54	−0.89	−0.83
OD_GCL-I	−1.23	−0.67	0.64	−4.48
OD_GCL_N	−1.74 *	−0.91	−2.74	−5.17
OD_GCL_T	−0.85	−6.44 *	−1.86	−4.39

Values were adjusted for age, gender, and mean arterial pressure. *p* < 0.05 is significant and shown with the * sign. Control is compared with the patient groups in the same regions. OD: right eye, C: central, S: superior, I: inferior, N: nasal, T: temporal, MT: macular thickness, RNFL: retinal nerve fiber layer thickness, and GCL: ganglion cell layer thickness. The control group is used as a reference cohort.

**Table 5:** Correlation between SCP vessel density and retinal layers thickness among the four groups.

Group	Central SCP VD and MT		Central SCP VD and GCL	
	r Value	*p*-Value	r Value	*p*-Value
Control	0.63	<0.01	0.72	<0.01
DM	0.41	0.03	0.56	<0.01
Mild NPDR	0.78	<0.01	0.78	<0.01
Moderate NPDR	0.84	<0.01	0.76	<0.01

GCL: ganglion cell layer thickness, NPDR: non-proliferative diabetic retinopathy, DR: diabetic retinopathy, SCP: superficial capillary plexus, VD: vessel density, and MT: macular thickness.

## Data Availability

Data are not available for public.
